# Supplementary material for: Developing Personas of Gamers with Problematic Gaming Behavior among College Students Based on Qualitative Data of Gaming Motives and Push–Pull–Mooring
Source: Int J Environ Res Public Health. 2023 Jan 1;20(1):798. doi: 10.3390/ijerph20010798 (PMC9819251; doi:10.3390/ijerph20010798)
Supplement: Supplementary file 1 [file ijerph-20-00798-s001.zip › File S1.pdf]

File S1. The 26 codes definition

	Code name	definition
#1	Seek walkthrough	Collecting game walkthroughs or other players' experiences or watching live stream of people playing games.
	e.g., *There's a section of "World of Warcraft" in PTT, and it's full of information such as how to equip your character or how to train it and so on. I study it even harder than making my school reports. (01_001_02)*	
#2	Pursue achievement	Pursuing advancement, proficiency or achievement to having fun on playing games.
	e.g., *Since I am really aggressive, I must win the last round before I go to bed, and that is also why I got addicted to the game. (04_002_03)*	
#3	Immerse in gaming	Getting so immersed in games that the focus and the state of mind stay completely on the game.
	e.g., *Remember the first time I met LOL and WOW. "Why on earth is there a game so fun to play?" Because this kind of feeling, I put the games at the first order then. (10_003_03)*	
#4	Spend money or time	Spending money on the game currency, monthly card or arranging certain amount of time to play based on the game rule.
	e.g., *Online games take charge. So I might just play them until the free term ends. That is, I only play them when they're free. (16_004_01)；Games cost monthly fee, so if you don't play them, it's your lost. That's why I hanged in the game even if I had nothing to do in it. (12_004_02)*	
#5	Prioritize gaming	Placing the games in the first order when arranging daily routine.
	e.g., *Actually, I think about the games in class or playing basketball from time to time. (10_005_01)*	
#6	Reserve a large and constant play time	Reserving constant and large amount of time for gaming.
	e.g., *We start gaming at 9:00pm every night in our junior year. It's 1 hour per round and if there was no class before 10 the next day we would play until 1 or 2 am. (01_006_01)*	
#7	Play games with acquaintances	Playing games with classmates, roommate, or friends.
	e.g., *We usually find acquaintances to play with if it's link play. (09_007_01)*	
#8	Play games with teammate through voice chat software	Playing online games with voice chat software to communicate with teammates.
	e.g., *I usually play with online friends. Interaction is pretty crucial for a good team work. We communicated through Skype when playing with others, which is easier to gain a victory. (17_008_01)*	
#9	Play games with friends at the Internet café	Going to the Internet café to play online games with friends.
	e.g., *Since my friends and I don't live together, it would be inconvenient if we want to play together while communicating in the same space. To meet our needs, we usually go to Internet café to play games. (20_009_01)*	
#10	Play games with roommates at dorm	Playing with roommates as a team in a game when gaming.
	e.g., *We can choose our roommates in sophomore. Because we all took a major in Chinese, we had really strong bonds. If there was a classmate who wanted to play but didn't know how to, we took him on and covered him. We would definitely cover him if asking him to play together. (01_10_01)*	
#11	Play with strangers	Playing online game with strangers.
	e.g., *(Is there any acquaintances among the people whom you play with?) There are some classmates but also some strangers since there aren't many people playing this particular game in the dorm. (17_011_01)*	
#12	Play games to have common topics to talk about	Play the same online game as classmates or friends do for having comment topics in chit chatting.
	e.g., *Thinking about what I really got from this game are memories and more chit chatting. (10_012_01)*	
#13	Spend long time on SNS	Spending a lot of time checking social website, viewing acquaintances' information or socializing with them.
	e.g., *The two websites on which I spent most of my time online is Facebook and MSN, which count for about 80% in total. I socialize and chat with people there. (20_013_01)*	
#14	Maintain the existing relationship	Interacting with people online mainly for keeping the bonds between friends.
	e.g., *Once I got used to tracking my friends on social websites, I get to know some fun stuff such as who went to Alishan or who went karaoke yesterday. (01_014_01)*	
#15	Have heart-to-heart talks with friends	Preferring talking about one's private feelings or listening to others on the social network.
	e.g., *When I was sad, I would go onto the Facebook or MSN to chat with my friends. (11_015_01)*	
#16	Run away	Escaping from the depression, boring and melancholy of real life and diving into

		games.
	e.g., *(So is the pressure released or not?) The stress was released temporarily, but right after the game finished a sense of guiltiness came back and the stress was there again. (04_016_01)*	
#17	Feel unsure about the future	Having no plans for future and feeling unsure, or having no goals and life focus while interviewees were senior.
	e.g., *My senior year is about to end and I'll be on my way to the military service. After that, I really don't know what to do. (01_017_01)*	
#18	Change game due to losing	Changing games to play due to the unvarying of game, losing novelty or limitation of personal ability.
	e.g., *I went into the world of online games officially in the sophomore year. But later I played less and less since I was a little stuck; I just couldn't get my level up anymore so I quit. (18_018_01)*	
#19	Be away from keyboard	Leaving the computer on so that one can enter the games or Internet quickly at any time.
	e.g., *I usually don't turn off my computer since the electricity and Internet fee are all free in the dormitory. (18_019_02)*	
#20	Be distracted while surfing the Net	Being unable to focus on one thing. One tend to do other things or switch from page to page while surfing the Net.
	e.g., *RPG games are relaxing since they're like chatting on MSN. They take simple moves which doesn't require the brain to work, so I can read novels, comics and chat with others while gaming. (12_020_01)*	
#21	Check with high frequency in a short time	Checking social network software for many times or uploading articles at high frequency but with short time.
	e.g., *I might go check Facebook once an hour. (01_021_01)*	
#22	Aim to reduce gaming	Having goals in real life such as taking tests to enter a new career, working part-time or serving as staffs in college clubs, resulting in the decrease of game time.
	e.g., *I used to work part-time in a restaurant named San-Huang-San-Jia in my freshman and sophomore years, which decreased my gaming time. (10_022_01)*	
#23	Aim to reduce surfing the Net	Having goals in real life such as taking tests to enter a new career, working part-time or serving as staffs in college clubs, resulting in the decrease of game time.
	e.g., *The club activities occupied most of my time in my sophomore, so I seldom use the Internet then. (03_023_01)*	
#24	Lose control	Being unable to restrict oneself from the urge of gaming and surfing the Net.
	e.g., *I tried to decrease the time of playing games before. But I just couldn't help but want to play. (09_024_01)*	
#25	Get negative consequences	Suffering from the uncomfortableness such as irregular eating, lengthy sit still, backache, or work/study-related bad consequences brought by surfing the Net or playing games.
	e.g., *I just couldn't get up before nine during the time I was addicted to playing games, so I failed a class due to super low attendance rate. (01_025_01)*	
#26	Self-aware of addiction	Being aware that oneself has been addicted to the Internet or games.
	e.g., *I might just keep using the Internet right after I'm back to the dorm and I am stuck there. (Is there anyone who has more serious symptoms than you do?) Nope, I think mine is one of the most serious. (18_026_01)*	
