# Supplementary material for: Developing Personas of Gamers with Problematic Gaming Behavior among College Students Based on Qualitative Data of Gaming Motives and Push–Pull–Mooring
Source: Int J Environ Res Public Health. 2023 Jan 1;20(1):798. doi: 10.3390/ijerph20010798 (PMC9819251; doi:10.3390/ijerph20010798)
Supplement: Supplementary file 1 [file ijerph-20-00798-s001.zip › File S2.pdf]

File S2. The four persona cases codes distributed

Topic	Theme	category	Sub category	Code name	4 persona cases			
					I-1	I-2	II-1	II-2
high-risk gamer’s push-pull-mooring	T1. Gaming motives push-pull effect	C1. Advancement	C11. level upgrade	CO01. Seek walkthrough	*			*
				CO02. Pursue achievement	*	*	*	*
				CO03. Immerse in gaming				*
			C12. Gamer’s routine	CO04. Spend money or time	*	*		
				CO05. Prioritize gaming		*	*	*
				CO06. Reserve a large and constant play time	*		*	*
		C2. Socializing	C21. Socialization through online gaming	CO07. Play games with acquaintances	*		*	*
				CO08. Play games with teammate through voice chat software	*			*
				CO09. Play games with friends at the Internet café			*	*
				CO10. Play games with roommates at dorm	*	*	*	
				CO11. Play with strangers			*	*
				CO12. Play games to have common topics to talk about	*			*
			C22. Socialization through SNS	CO13. Spend long time on SNS	*	*		
				CO14. Maintain the existing relationship	*		*	
				CO15. Have heart-to-heart talks with friends				*
		C3. Escapism	C31. Reason for escaping	CO16. Run away			*	
				CO17. Feel unsure about the future	*	*	*	*
				CO18. Change game due to losing	*	*		
			C32. Action of escaping	CO19. Be away from keyboard		*		*
				CO20. Be distracted while surfing the Net	*	*		
				CO21. Check with high frequency in a short time	*	*	*	
	T2. Gaming consequences mooring effect	C4. Shift focus to real-life		CO22. Aim to reduce gaming	*		*	*
				CO23. Aim to reduce surfing the Net	*		*	
		C5. Symptoms of addiction		CO24. Lose control	*	*		*
				CO25. Get negative consequences	*		*	*
				CO26. Self-aware of addiction	*	*	*	
